# Mechanically activated catalyst mixing for high-yield boron nitride nanotube growth

**DOI:** 10.1186/1556-276X-7-417

**Published:** 2012-07-24

**Authors:** Ling Li, Lu Hua Li, Ying Chen, Xiujuan J Dai, Tan Xing, Mladen Petravic, Xiaowei Liu

**Affiliations:** 1 MEMS Center, Harbin Institute of Technology, Harbin 150001, China; 2 Institute for Frontier Materials, Deakin University, Geelong Waurn Ponds Campus, Waurn Ponds, Victoria 3216, Australia; 3 Department of Physics and Center for Micro and Nano Sciences and Technologies, University of Rijeka, Rijeka 51000, Croatia

**Keywords:** Boron nitride nanotube, Mechanical milling, Nanostructured materials, Synthesis, X-ray absorption fine structure, PACS, 81.07.De, 81.16.Be, 68.55.A-.

## Abstract

Boron nitride nanotubes (BNNTs) have many fascinating properties and a wide range of applications. An improved ball milling method has been developed for high-yield BNNT synthesis, in which metal nitrate, such as Fe(NO_3_)_3_, and amorphous boron powder are milled together to prepare a more effective precursor. The heating of the precursor in nitrogen-containing gas produces a high density of BNNTs with controlled structures. The chemical bonding and structure of the synthesized BNNTs are precisely probed by near-edge X-ray absorption fine structure spectroscopy. The higher efficiency of the precursor containing milling-activated catalyst is revealed by thermogravimetric analyses. Detailed X-ray diffraction and X-ray photoelectron spectroscopy investigations disclose that during ball milling the Fe(NO_3_)_3_ decomposes to Fe which greatly accelerates the nitriding reaction and therefore increases the yield of BNNTs. This improved synthesis method brings the large-scale production and application of BNNTs one step closer.

## Background

Boron nitride nanotubes (BNNTs) are a promising nanomaterial with many fascinating properties and a wide range of applications. BNNTs have high thermal and chemical stabilities [1,2] and can be reinforced into composites working in harsh environments. BNNTs have a wide bandgap close to 6 eV and strong deep ultraviolet light emission [3,4], useful in fabricating optoelectronic devices at nanoscale. Transistor behaviour has been predicted from BNNTs due to their giant stark effect [5]. In addition, BNNTs have many potential bioapplications, including drug delivery, nanofluidics and nanoscaled biosensors [6-8].

BNNTs were first produced using arc discharge and laser ablation methods [9,10]. Later on, other synthesis routes, including ball milling and annealing [11-14], chemical vapour deposition [15-17] and other thermal chemical methods [6,18], were demonstrated. The ball milling and annealing method has been shown to be able to produce larger quantities of BNNTs and more easily to scale up [11-13]. In this process, metal nanoparticles from repeated collisions between milling jar and balls during ball milling act as catalysts for BNNT growth [11-13]. The recent B ink process added metal nitrate (e.g. Fe(NO_3_)_3_ or Co(NO_3_)_2_) to the milled B powder in the form of ethanol solution, and the additional nitrate catalyst showed a more efficient catalytic effect and greatly boosted the growth of BNNTs [19,20], especially in the case of the growth of BNNT thin films on different surfaces [21].

Here, we report an improved ball milling and annealing method for BNNT synthesis, which shows a better BNNT yield compared to the original ball milling method as well as the B ink method. The method involves ball milling of metal nitrate with B powder, and the subsequent thermal annealing of the milled powder in nitrogen-containing gases grows a high density of BNNTs.

## Methods

Amorphous B powder (95% to 97%, Fluka, Sigma-Aldrich Corporation, St. Louis, MO, USA) and 10 wt.% Fe(NO_3_)_3_•9H_2_O (98%, Pronalys, Thermo Fisher Scientific, Waltham, MA, USA) were sealed in a stainless milling jar with several hardened steel balls. The weight ratio of ball to powder was 80:1. Dehydrated NH_3_ gas was filled into the jar at a pressure of 250 kPa. The ball milling was conducted on a custom-built vertical milling machine and lasted for 150 h at a speed of 110 rpm at room temperature. The milled powder was then heated up to 1,100°C in N_2_ + 15% H_2_ or 1,300°C in NH_3_ for 3 to 6 h to produce BNNTs.

The morphology and chemical composition of the products were investigated using a Supra 55VP scanning electron microscope (SEM; Carl Zeiss AG, Oberkochen, Germany) equipped with an energy dispersive X-ray spectroscopy (EDX) system. A JEOL-2100 transmission electron microscope (TEM; JEOL Ltd., Akishima, Tokyo, Japan) was used to check the structure of the nanotubes. The material phases were analysed by X-ray diffraction (XRD; PANalytical B.V., Almelo, The Netherlands). The nitriding reaction rates of different samples were compared by thermogravimetric analyses (TGA; Netzsch, Hanau, Germany) which monitored the sample weight changes up to 1,300°C (with a temperature increasing rate of 10°C/min) in a pure N_2_ atmosphere. A Thermo Fischer Scientific K-alpha X-ray photoelectron spectroscopy (XPS) system was used to measure the chemical compositions of the milled B powders. Pass energies of 100 and 20 eV were used in survey and high-resolution scans. All XPS data were corrected using the binding energy of C-C at 284.6 eV. The near-edge X-ray absorption fine structure (NEXAFS) measurements were performed in the ultrahigh vacuum chamber (approximately 10^−10^ mbar) at the undulator soft X-ray spectroscopy beamline of the Australian Synchrotron, Victoria, Australia. The raw NEXAFS data were normalized to the photoelectron current of the photon beam, measured on an Au grid.

## Results and discussion

**Figure 1:**
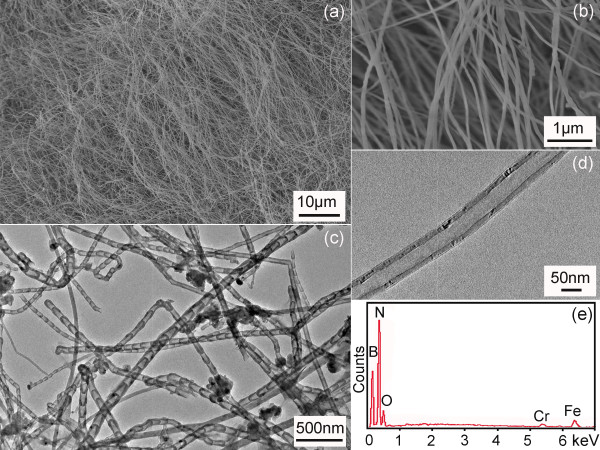
**SEM images (a,b), TEM images (c,d) and EDX spectrum (e) of BNNTs.** The BNNTs were produced by heating the Fe(NO_3_)_3_ + B milled powder in N_2_ + 15% H_2_ gas at 1,100°C.

**Figure 2:**
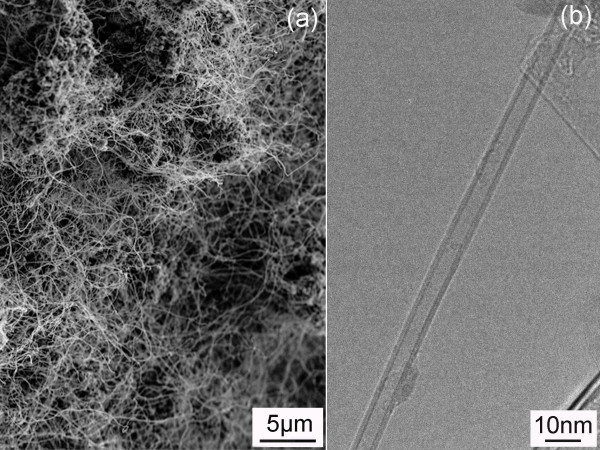
**SEM (a) and TEM (b) images of the BNNTs produced in NH**_**3**_**gas at 1,300°C.**

Figure 1a,b shows SEM images of a typical product after the heating of the milled powder in N_2_ + 15% H_2_ atmosphere at 1,100°C. The nanotubes mainly have diameters of 60 to 100 nm and lengths of 50 to 200 μm. Majority of the tubes have a bamboo-like structure (Figure 1c), but cylindrical nanotubes can also be easily found under TEM (Figure 1d). The BNNTs normally have metal catalysts at the tips, suggesting that the BNNTs produced in the current method are following the same vapour-liquid-solid (VLS) growth mechanism as in the original ball milling method and the B ink method [11,19,22]. The EDX result (Figure 1e) confirms that the sample mainly consists of B and N elements, along with O impurities and metal catalysts from the added Fe(NO_3_)_3_ as well as the repeated collisions between the steel milling jar and balls during ball milling [11,13]. Cylindrical BNNTs with smaller diameters of approximately 10 nm can be harvested if the milled powder is heated in NH_3_ gas at 1,300°C (Figure 2a). These BN tubes have shorter lengths of 5 to 8 μm. The TEM image in Figure 2b shows the straight walls of a BNNT produced under this condition.

**Figure 3:**
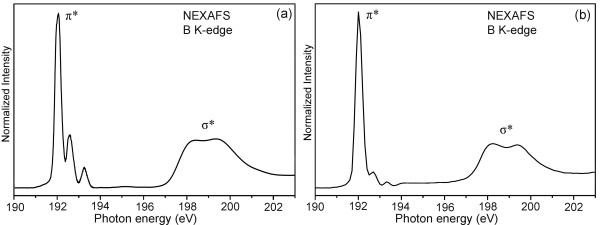
**NEXAFS spectra around the B K-edge region of BNNTs produced from heating the milled powder.** In (**a**) N_2_ + 15% H_2_ at 1,100°C and (**b**) NH_3_ at 1,300°C.

The precise chemical structure and bonding of the produced BNNTs were determined by NEXAFS spectroscopy. In the B K-edge region, the BNNTs produced in N_2_ + 15% H_2_ and NH_3_ gases mainly show similar NEXAFS spectra (Figure 3a,b): a sharp π* resonance at 192.0 eV and broad σ* resonances at higher energies, corresponding to core-level electron transitions to the unoccupied antibonding *s + p*_*z*_ and *p*_*x*_ *+ p*_*y*_ orbitals due to the dipole selection rule Δ*l* = ±1, where Δ*l* is the angular momentum quantum number difference between the initial and final states [23,24]. The strong π* resonances from both types of BNNTs suggest that B and N atoms have a covalent *sp*^*2*^ bonding. Close to the strong π* resonance, there also exist two satellite peaks at 192.6 and 193.3 eV, commonly observed from various hBN materials [25-28]. These weak peaks represent point defects in the form of one or two nitrogen vacancies decorated by oxygen atoms in a hexagonal ring [24,29]. The relatively stronger satellite peaks shown in the NEXAFS spectrum of the N_2_ + 15% H_2_-produced BNNTs (Figure 3a) indicate more point defects and chemical impurities in the product, consistent with their bamboo structure. In contrast, the small cylindrical BNNTs synthesized in NH_3_ gas have much better structure and purity. In addition, the full width at half maximum of the π* resonance of the NH_3_-synthesized BNNTs (0.348 eV) is slightly smaller than that of the N_2_ + 15% H_2_-synthesized BNNTs (0.368 eV), suggesting slightly more disorders in the latter product.

**Figure 4:**
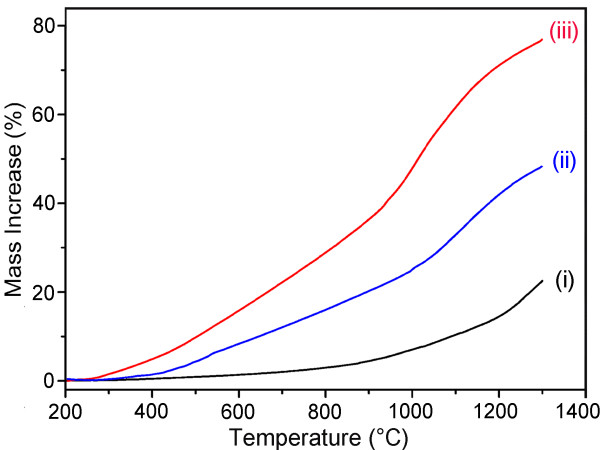
**TGA curves.** TGA curves of (i) B powder ball milled alone, (ii) B ink with 0.04 M Fe(NO_3_)_3_ ethanol solution and (iii) B powder ball milled with 0.04 M Fe(NO_3_)_3_, heated up to 1,300°C in N_2_ gas with a temperature increasing rate of 10°C/min.

The improved BNNT yield by mechanically milling B powder and Fe(NO_3_)_3_ was verified by TGA. Figure 4 compares the mass increases, implying the level of nitriding and BNNT formation, among three samples during the heating in a N_2_ atmosphere: (i) B powder milled alone, as in the original ball milling and annealing method [11,13]; (ii) B ink, a mixture of ball milled B powder and Fe(NO_3_)_3_ ethanol solution, as in the case of the B ink method [19]; and (iii) milled powder of B and Fe(NO_3_)_3_ mixture. In samples (ii) and (iii), the same amount (0.04 M) of Fe(NO_3_)_3_ was added. Among the three samples, the B powder milled alone (i) had the lowest mass gains of only 10.6% at 1,100°C and 22.4% at 1,300°C and therefore the slowest nitriding rate. The B ink sample (ii) showed a dramatic increase in the BN formation rate during heating, evidenced by 32.3% and 48.1% weight gains at 1,100°C and 1,300°C, respectively. This increase was mainly due to the catalytic enhancement from the Fe(NO_3_)_3_ added in the form of nitrate ethanol solution, which has been carefully studied previously [19]. The highest mass gain was from the Fe(NO_3_)_3_ and B milled powder (iii), which showed 62.1% and 76.5% weight increases at 1,100°C and 1,300°C, respectively. In addition, the sample (iii) had the lowest starting nitriding temperature among the three samples. Note that the TGA results shown here should not be directly compared with those in [19] because N_2_ instead of N_2_ + 5% H_2_ was used in the current study (hydrogen can further enhance BN formation) [30]. These results clearly illustrate that the improved milling method has even higher BNNT yield than the B ink method.

**Figure 5:**
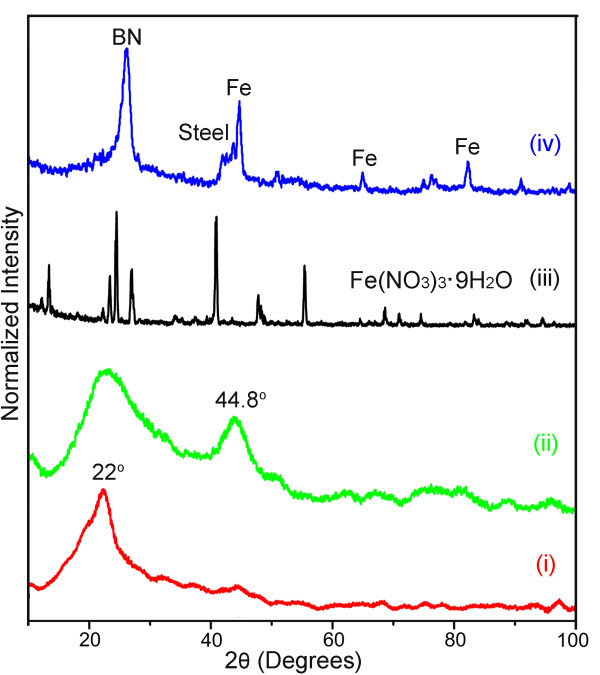
**XRD patterns.** XRD patterns of (i) B powder milled alone, (ii) B powder milled with Fe(NO_3_)_3_, (iii) as-purchased Fe(NO_3_)_3_•9H_2_O and (iv) BNNTs produced by the improved ball milling method.

XRD analyses were used to investigate why the milled catalyst had a higher efficiency in BNNT production. Figure 5 (i) shows the XRD pattern of the B powder milled alone: a broad diffraction peak centred at approximately 22°, possibly representing an amorphous B phase caused by extensive milling. When Fe(NO_3_)_3_ was added to the milling, this peak further broadened, and an additional peak at 44.8° appeared (Figure 5 (ii)). Because the only difference between the two milled powder is the addition of Fe(NO_3_)_3_, the additional peak at 44.8° should be Fe(NO_3_)_3_ related. However, this peak does not match Fe(NO_3_)_3_ whose XRD pattern is shown in Figure 5 (iii) for comparison. This suggests that the Fe(NO_3_)_3_ may have decomposed during the milling, which actually is not surprising because Fe(NO_3_)_3_ is not stable and can easily decompose to iron oxide under the high-energy ball milling collisions [31,32]. However, the 44.8° peak position is more close to the Fe peak at 45.1° or Fe_2_B peaks at 42.6° and 45.0°. Both Fe and Fe_2_B possibilities are reasonable because the iron oxide decomposed from Fe(NO_3_)_3_ could be further reduced to Fe by ammonia gas or B powder, and it is also possible that the iron oxide has a disproportionation reaction with the excessive B to form metastable amorphous boron iron nanoparticles during milling (that is, iron is reduced from iron oxide by B and simultaneously reacts with B to form boron iron) [33]. Although no research on the ball milling of B and Fe(NO_3_)_3_ has been conducted, there are reports on the milling of B and Fe, in which FeB and Fe_2_B were formed [34,35]. Unfortunately, it is hard to judge from the XRD results alone on which possibility is the case in our experiment because of the amorphization and crystal size reduction induced broadening of the XRD peaks after milling.

**Figure 6:**
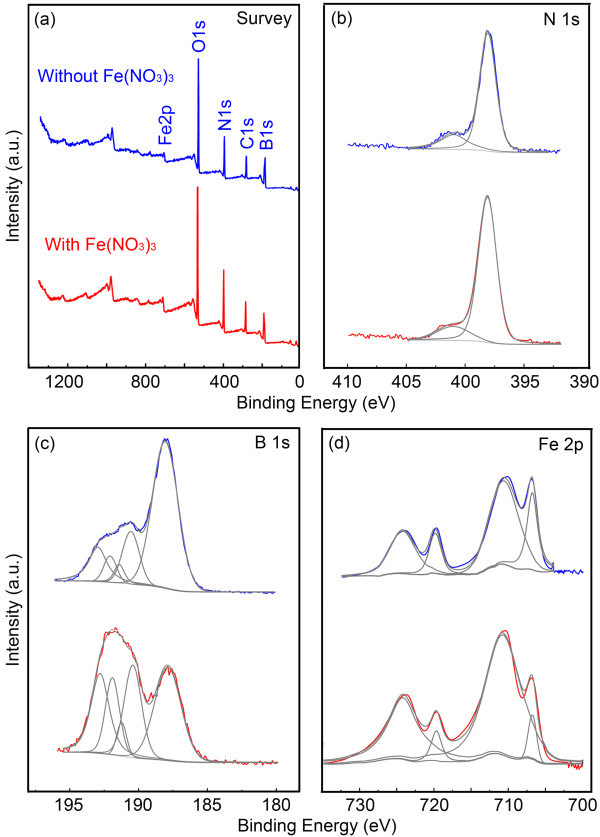
**XPS spectra and corresponding fittings of B powder milled with (red) and without (blue) Fe(NO**_**3**_**)**_**3**_**.** (**a**) Survey scans, (**b**) N 1s region, (**c**) B 1s region and (**d**) Fe 2p region.

Therefore, XPS was used to get more information on the B powder milled with and without Fe(NO_3_)_3_. Figure 6a compares the XPS survey scans on the two powders. The high level of O is due to the surface oxidation of the chemically reactive B particles produced by milling when exposed to air. It is interesting that high contents of N have been detected from both the milled powders. In contrast, the un-milled B powder had almost no N signal. The high resolution scans in N 1s region (Figure 6b) reveal that the milling-introduced N was mainly in the form of B-N bonds, as evidenced by the dominating peaks centred at 397.9 eV. The N mainly came from the NH_3_ milling atmosphere rather than the added Fe(NO_3_)_3_ because (1) B powder milled alone also resulted in high N contents (Figure 6b) and (2) 10 wt.% of Fe(NO_3_)_3_•9H_2_O was added to B powder, which only gave a maximum B:N ratio of 1:0.0083, enormously less than the detected B:N ratios: 1:0.36 for the B + Fe(NO_3_)_3_ milled powder and 1:0.21 for B milled alone powder. It reveals that the milling of Fe(NO_3_)_3_ injected 71% more N to the B powder. This enhancement may come from the Fe decomposed from Fe(NO_3_)_3_, as Fe is a well-known catalyst in nitriding as well as decomposition of NH_3_[36]. Turning back to the XRD results in Figure 5 (i) and (ii), we realize that the approximately 22° diffraction peaks are partly contributed by the BN (002) peak at 26.8°, and the further broadening of the 22° peak after the addition of Fe(NO_3_)_3_ is due to the formation of more nitrides, leading to a stronger XRD signal of BN. According to XPS, no NO_3_^−^ peak (at 406.8 eV) is shown in the N 1s region from the Fe(NO_3_)_3_ milled powder, indicating that all Fe(NO_3_)_3_ were possibly decomposed during ball milling. This is consistent with the XRD results in Figure 5. The B 1s spectra (Figure 6c) consist of a series of peaks: 192.8 eV (B_2_O_3_), 191.9 eV (B_x_O_y_), 191.3 eV (BN_x_O_y_), 190.3 eV (BN) and 188 eV (B-B). This suggests that the 22° XRD peak may also contain the signals from boron oxides. Nevertheless, it should also be noted that XRD and XPS analyses have totally different measuring depths (microns for XRD and a few nanometers for XPS) and therefore may provide different information from the surface and bulk of a sample. Again, the BN peak is stronger for the Fe(NO_3_)_3_ milled powder, confirming that much more nitriding happened when Fe(NO_3_)_3_ was added. However, due to the overlapping between the Fe_2_B peak at 188.2 eV and the B-B peak at 188 eV, it is hard to judge the possible existence of Fe_2_B from the B 1s spectra. In the Fe 2p region (Figure 6d), both the milled powders show Fe peaks at 706.8 and 720.0 eV and Fe_2_O_3_ peaks at 710.7 and 724.3 eV. The binding energy of Fe_2_B is 706.7 eV, overlapping with the Fe 2p_3/2_ peak at 706.8 eV, which is not helpful to directly determine the presence of Fe_2_B. However, the 706.8 eV peak from the Fe(NO_3_)_3_ milled B powder is not more intense than that of the B milled alone powder, which is contradictory to the existence of Fe_2_B. So, it is more likely that the final state of Fe(NO_3_)_3_ after ball milling is Fe. This conclusion is consistent with (1) 71% more B-N bonds formed when Fe(NO_3_)_3_ is added, because Fe has a strong catalytic effect in nitriding and ammonia decomposition; and (2) the more level of Fe_2_O_3_ (710.7 and 724.3 eV) when Fe(NO_3_)_3_ is added to ball milling, because the Fe decomposed from Fe(NO_3_)_3_ can be easily oxidized to Fe_2_O_3_ on the surface after exposed to air.

The above analysis results reveal the following enhancing mechanism of ball milling Fe(NO_3_)_3_ with B powder. During the milling, Fe(NO_3_)_3_ was first decomposed to iron oxide under milling collisions, and the iron oxide was then reduced to Fe by either NH_3_ or B. These reduced Fe was much more reactive than the steel particles from the collisions between the steel milling jar and balls, due to both the more stable nature of steel and the smaller particle sizes of the chemically reduced Fe. During the heating up to 1,300°C in nitrogen-containing gases, the Fe became quasi-liquid at lower temperature than the steel particles and provides a better catalytic effect. BNNTs started to form from the BN layers precipitating on the surface of the metal particles diffused with excessive B and N atoms, following the VLS growth mechanism [22]. As a result, a strong BN phase XRD peak was observed after the heating (Figure 5 (iv)). The Fe(NO_3_)_3_ + B milled powder showed even better BNNT yield than the B ink, possibly thanks to the 71% more N content of the Fe(NO_3_)_3_ milled powder. The amorphous B-N phase produced by milling is unstable and can directly provide N source for BNNT growth with little aid from the nitrogen-containing gas. This can reduce the temperature of BNNT formation and result in a better yield of BNNTs.

## Conclusions

The ball milling of amorphous B powder and metal nitrate, such as Fe(NO_3_)_3_, produces a more effective precursor for BNNT production. The detailed XRD and XPS investigations reveal that during the milling of B powder and Fe(NO_3_)_3_, Fe(NO_3_)_3_ is first decomposed to iron oxide which is further reduced to Fe. The reduced Fe greatly increases the nitriding reaction of the B powder during ball milling in ammonia (NH_3_) atmosphere, evidenced by 71% more N content. The pre-formed amorphous BN is much more reactive than crystallined hBN and can directly provide N source for the formation of BNNTs; therefore it lowers the BNNT formation temperature and improves the nitriding rate. The homogeneously mixed additional Fe reduced from Fe(NO_3_)_3_ also acts as effective catalysts and assists the growth of BNNTs. As a result, a higher yield of BNNTs can be synthesized. Other metal nitrates, such as Mg(NO_3_)_2_ and Co(NO_3_)_2_, can also be used in this method.

## Competing interests

The authors declare that they have no competing interests.

## Authors' contributions

LL produced the materials; conducted the SEM, XRD, EDX and TGA measurements; and drafted the manuscript. LHL provided the idea, designed this study, carried out the TEM and NEXAFS investigations and drafted the manuscript. YC provided the idea and drafted the manuscript. XJD did the XPS analyses. TX assisted in the ball milling process. MP participated in the NEXAFS measurements. XL participated in the manuscript preparation. All authors read and approved the final manuscript.
